# Novel Synthesis of Slightly Fluorinated Graphene Quantum Dots with Luminescent and Paramagnetic Properties through Thermal Cutting of Fluorinated Graphene

**DOI:** 10.3390/ma11010091

**Published:** 2018-01-08

**Authors:** Qian Feng, Wenqing Xiao, Yuan Liu, Yongping Zheng, Yuda Lin, Jiaxin Li, Qingying Ye, Zhigao Huang

**Affiliations:** 1 Fujian Provincial Key Laboratory of Quantum Manipulation and New Energy Materials, College of Physics and Energy, Fujian Normal University, Fuzhou 350117, China; wqxiao1995@163.com (W.X.); zyp@fjnu.edu.cn (Y.Z.); linyuda1993@163.com (Y.L.); lijiaxin@fjnu.edu.cn (J.L.); qyye@fjnu.edu.cn (Q.Y.); 2 Fujian Provincial Collaborative Innovation Center for Optoelectronic Semiconductors and Efficient Devices, Xiamen 361005, China; 3 Faculty of Science, Jiangsu University, Zhenjiang 212013, China; yliu@ujs.edu.cn

**Keywords:** fluorinated graphene, thermal cutting, graphene quantum dots, photoluminescence, paramagnetism

## Abstract

A novel approach has been developed to synthesize slightly fluorinated graphene quantum dots (GQDs-F) through thermal cutting of highly fluorinated graphene. The fluorinated graphene with substantial structure defects is fragile and is readily attacked. The direct evaporation of abundant CF_n_ (*n* = 2, 3) groups near structure defects lead to the loss of adjacent skelton C atoms, and the fluorinated graphene can be thermally cut into GQDs-F with a relatively uniform nanosize in pyrolysis at 810 K. The GQDs-F with a low F/C atomic ratio of ca. 0.03 exhibit excitation wavelength-dependent properties with multicolor photoluminescence (PL) from blue to green. At the same time, F adatoms that are most likely located at the edges of GQDs-F have a high efficiency of introducing paramagnetic centres, and GQDs-F show a strong paramagnetism because of sp^3^-type defects and magnetic zigzag edges. The graphene quantum dots with such multimodal capabilities should have great applied value in material science.

## 1. Introduction

Graphene quantum dots (GQDs) present outstanding photoelectric and magnetic properties due to quantum confinement and unique edge effects, which shows many potential applications in biomedical field, photovoltaic devices, spintronic devices, etc. [1,2,3,4,5,6]. As an ideal fluorescent material, GQDs from various preparing methods show different photoluminescence (PL) colors in the visible range, and it is influenced by various parameters, such as lateral size [7,8] and functional groups [9,10,11]. In addition, the magnetism of GQDs, because of high edge-to-area ratio and abundant spin-polarized zigzag edge states [12,13,14], has aroused tremendous interest. Although there is predicted intriguing magnetism, however, only a few studies have experimentally revealed the magnetic properties of GQDs [2,15,16]. The magnetic measured results show most of the GQDs derived by oxidative cutting are nonmagnetic because of the saturated carboxylation of GQDs’ edges, and only few are paramagnetic [15]. As reported, the doping of graphene by light element can locally break the delocalized π bonding network of the bipartite graphene lattice, and are considered to be good routes to induce magnetic moments [17,18,19,20]. Thus, to introduce sp^3^-type defects or magnetic zigzag edges in the GQDs is of significance to obtain high magnetization. The doping GQDs with light elements, such as fluorine or boron, were successfully synthesized, and the fluorescence properties were modulated [2,21,22,23]. However the influence on magnetic properties has seldom been studied [2]. Strong paramagnetic properties, as well as excellent fluorescence, are regarded as basic factor of GQDs in some pratical applications, such as good contrast agents for safe magnetic resonance imaging [2].

Recently, a variety of approaches have been developed to synthesize GQDs, including top-down and bottom-up methods [23,24,25,26,27]. The predominant top-down methods involving hydrothermal [21,22,24], microwave [23], electrochemical methods [25], etc. The chemical cutting of carbon materials was always carried out by using strong oxidants, and it is difficult to remove the by-products, limiting the large scale preparation of GQDs. In particular, the high oxidation level of the producing GQDs hinders the exploring of the structural origin of their PL properties [9,10] and suppresses the edge states magnetism [15]. Some effort has been taken to develop precise bottom-up approaches to produce pristine GQDs from aromatic structures of molecules [26,27]. The properties of the final products can be precisely controlled, but their complicated multi-step preparation also limits their application [26,27]. On the whole, there is a practical demand to develop an effective method for synthesizing convenient GQDs with excellent photoelectric and magnetic properties.

Herein, we report novel synthesis of slightly fluorinated graphene quantum dots with luminescent and paramagnetic properties through thermal cutting of fluorinated graphene. Although perfect graphene fluoride is known as a two-dimensional counterpart of Teflon [28], the prepared fluorinated graphene in this work with high percent of structure defects is fragile [29]. Strikingly, we found that the CF_n_ (*n* = 2, 3) groups near structure defects are like scissors in thermal defluorination, which can evaporate as C_2_F_4_ product and greatly enlarge the structure defects of fluorinated graphene [30,31]. The fluorinated graphene can be thermally cut into GQDs-F with a low F/C atomic ratio of ca. 0.03 in pyrolysis at 810 K. The novelty synthesis is convenient and effective, which has a high yield of 13.2%. The GQDs-F shows an intrinsic blue emission, and exhibits an excitation wavelength-dependent property, emitting multicolor PL from blue to green. Intriguingly, it is found that the GQDs-F show spin-half paramagnetism, and the number of localized spins of GQDs-F is high, up to ca. 7.33 × 10^19^ g^−1^, because of the doping of fluorine and unreconstructed zigzag edges. The prepared GQDs-F with luminescent and paramagnetic properties can be used as a multimodal material in biomedical fields, or other practical fields.

## 2. Materials and Methods

### 2.1. Materials

All chemicals were used as received. Natural flake graphite powder (500 mesh), NaNO_3_, KMnO_4_, and condensed H_2_SO_4_ were bought from Alfa Aesar (Shanghai, China).

### 2.2. Sample Fabrication

Graphene oxide (GO) was synthesized by a modified Hummers’ method. To exclude the presence of impurities, the GO sample was washed with hydrochloric acid seven times and then with alcohol 10 times. Thereafter, the fluorinated graphene (FG) was prepared by directly heating the mixture of GO and XeF_2_ with a mass ratio of 1:13 in Teflon container at 200 °C for 30 h under Ar atmosphere. The FG powder was then separated into batches and annealed at different temperatures (780 K or 810 K) for 1 h under Ar atmosphere. After being cooled quickly to room temperature, the annealed samples were obtained, and were stored in nitrogen atmosphere.

### 2.3. Characterization Techniques

The morphologies of the samples were detected by transmission electron microscope (TEM, Tecnai G2 F20, Hillsboro, OR, USA) with the equipment operated at an accelerating voltage of 200 kV. The Raman spectra were tested using a LABRAM-HR micro-Raman system (Longjumeau, Paris, France) with a laser source of 532 nm. The hydrodynamic diameter was measured through dynamic light scattering (DLS) in dimethyl sulfoxide using the Malvern Zetasizer Nano ZS system (Malvern, Worcestershire, UK) with a He–Ne laser (633 nm, 4 mW). The X-ray photoelectron spectra (XPS) were tested on PHI5000 Versaprobe (ULVAC-PHI, Chigasaki, Kanagawa, Japan) by using Al Ka radiation. The absorption and fluorescence spectra were tested at room temperature on ultraviolet spectrophotometer (Shimadzu UV-3600, Kyoto, Japan) and fluorescence spectrophotometer (Shimadzu, RF-5301PC, Kyoto, Japan), respectively. The magnetic properties of the samples were measured by using a SQUID magnetometer, with a sensitivity of less than 10^−8^ emu (Quantum Design MPMS–XL, San Diego, CA, USA). The magnetic impurity elements of all the samples after magnetic test were tested by inductively coupled plasma analysis (ICP, Jarrell–Ash JA-1100, Franklin, MA, USA).

### 2.4. Quantum Yield Calculations

The quantum yield (QY) of GQDs-F was measured by using quinine sulfate (QY = 0.577) as a reference standard. The QY of GQDs-F in water was calculated according to

(1) $$\varphi =\varphi_{r}\left(I/I_{r}\right)\left(A_{r}/A\right)\left(n^{2}/n_{r}{}^{2}\right)$$

where *φ* is the quantum yield, *I* is the measured integrated emission intensity, *A* is the gradient from the plot of integrated fluorescence intensity versus absorbance, and *n* is the refractive index of the solvent (1.33 for water). The subscript ‘*r*’ refers to the standard [11]. 

## 3. Results and Discussion

**Figure 1:**
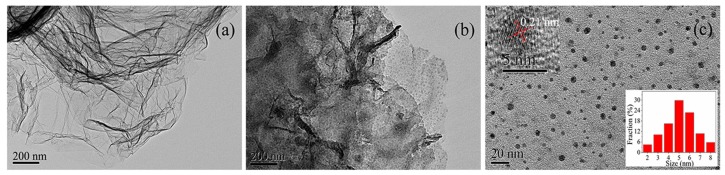
Transmission electron microscope (TEM) images of (**a**) FG; (**b**) the annealed sample fluorinated graphene (FG)-780, which was obtained by annealing FG at 780 K; (**c**) the annealed sample graphene quantum dots (GQDs)-F, which was obtained by annealing FG at 810 K. The upper left inset is HRTEM image, and the lower right inset is the diameter distribution of GQDs-F.

Figure 1 illustrates the typical TEM images of FG, FG-780 (annealed at 780 K), and GQDs-F (annealed at 810 K). From Figure 1a, it is found that FG has micrometer-sized flexible structure with many ripples, like a crumpled thin paper. Strikingly, as shown in Figure 1b, after being annealed at 780 K, most of the graphene nanosheets show distinct pyrolysis, decomposing into a great many relatively uniform graphene nanodots. These graphene nanodots are still attached together, keeping graphene in shape. Figure 1c shows the typical TEM image of the sample GQDs-F. It is found that the graphene nanosheets thoroughly decompose into scattered grahene quantum dots when annealed at a higher temperature of 810 K. The upper left inset is the HRTEM image with an in-plane (0–110) lattice spacing of 0.21 nm [21], indicating the high crystallinity of GODs-F. The corresponding sizes and hydrodynamic diameters extracted from TEM and DLS analyses are given in the lower right inset of Figure 1c and Figure S1 (Supplementary Materials), respectively, which demonstrate the small and relatively uniform sizes of the QGDs-F. The average diameter extracted from TEM is ca. 5.11 nm, which is smaller than the hydrodynamic diameter (~6.64 nm). It may be due to different surface states of the samples under different measured conditions [32]. Certain aggregation in the solvent may not be entirely avoided, which can also lead to the observed larger value of hydrodynamic size. 

**Figure 2:**
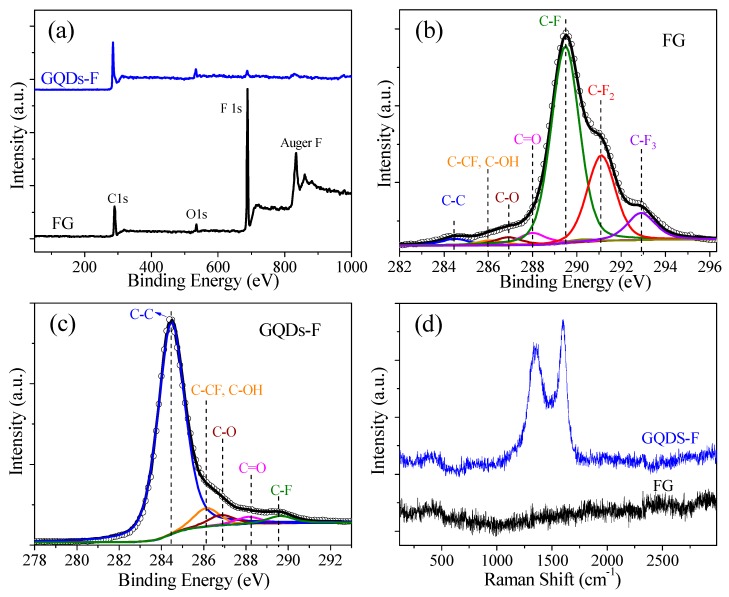
(**a**) The survey X-ray photoelectron spectra (XPS) spectra of FG and GQDs-F samples; (**b**) The C 1s XPS of FG, which were fine-scanned and deconvoluted into several components; (**c**) The C 1s XPS of GQDs-F, which were fine-scanned and deconvoluted into several components; (**d**) The Raman spectra of FG and GQDs-F.

Figure 2a gives the survey XPS of FG and GQDs-F. For FG, there exist two prominent peaks at 688.8 and 833.8 eV corresponding to photoemission of the F 1s core level and Auger electron [31,33]. After being annealed at 810 K, it is seen from the survey XPS of GQDs-F sample that the F signals decrease obviously, along with the increasing C 1s signals at ca. 284.5 eV. The atomic percents of the samples can be derived from the quantitative analysis of the XPS. The F/C atoms ratio of FG is ca. 1.30 for FG, which is larger than 1.0, indicating that the existence of CF_n_ (*n* = 2, 3) bonds. The F/C atoms ratio is obviously declined to ca. 0.03 for GQDs-F, revealing that thermal annealing can greatly induce the defluorination, and the obtained GQDs-F are slightly fluorinated GQDs. 

Figure 2b,c gives the typical C 1s XPS spectra of FG and GQDs-F, which were fine-scanned and deconvoluted into several components. The locations of C 1s XPS peaks corresponding to different chemical groups are also given in Table 1 [31,33]. When compared to FG, GQDs-F show an obvious increase in the percent of sp^2^ C–C bonds, as well as a decrease in the intensity of the CF_n_ peaks, manifesting the reformation of sp^2^ bonds and an evident defluorination. The contents of chemical groups of the samples determined from the deconvolution of C 1s spectra are shown in Table 1. Notably, for the FG sample, there is a non-negligible formation of CF_n_ (*n* = 2, 3) bonds (CF_2_: 23.9%, terminal CF_3_: 8.4%), generally located at graphene defects and edges [31,33,34]. It owes to the violent reaction of the direct fluorination of GO with excess of XeF_2_ at the high fluorination temperature, and oxygenic groups in GO greatly accelerate fluorination reaction by activating the adjacent aromatic regions and participating in the substitution reaction with fluorine radicals [34]. The component of oxygen containing groups of FG mainly assign to carboxyl (4.4%), aliphatic C–O groups (2.9%), and a few hydroxyls groups, indicating that oxygenic groups are easily replaced by fluorine, especially hydroxyls and carbonyls groups [33]. For GQDs-F, the percent of C–F bonds is 3.0%, which indicates that some strongly bonded fluorine can still persist after heating to 810 K. When compared to FG, the percent of oxygen containing groups is only slightly changed, implying that the oxygenic groups have a high stability in thermal annealing. Figure 2d gives the Raman spectra of FG and GQDs-F. For the FG sample, it is seen that there is no discernable Raman signature in Raman spectra, which is similar with that of highly fluorinated graphene, as previously reported [28]. After annealing, the Raman data of the annealed samples show obvious G peaks (at 1600 cm^−1^), corresponding to graphene recovery. The appearance of characteristic disorder–induced peak (D peak) at 1350 cm^−1^ with a high *I_D_*/*I_G_* (the ratio of the D peak to G peak) of 0.84, implies that the GQDS have high–concentration edge defects. 

**Figure 3:**
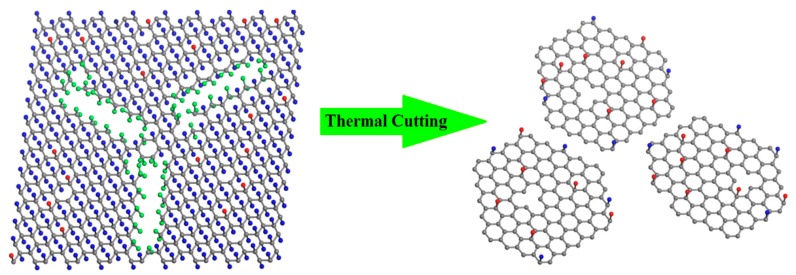
Schematic illustrations for GQDs-F through a novel thermal cutting of fluorinated graphene. The **left** is FG with a number of structure defects, characterizing by a high percent of C–F_n_ (*n* = 2, 3) bonds. The bonded F atoms of C–F_n_ (*n* = 2, 3) groups are highlighted by green balls, and other F adatoms are shown as blue balls. Carbon skeleton atoms and O-contained groups are shown as grey and red balls, respectively. When the annealing temperature is high enough, FG can be completely cut into scattered graphene quantum dots (**Right**).

**Figure 4:**
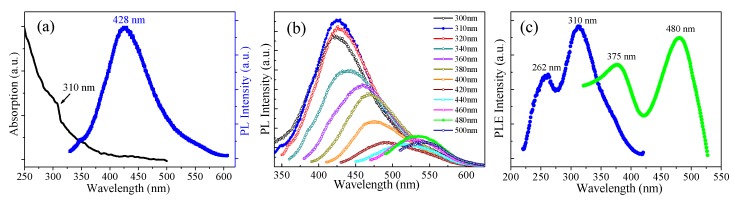
(**a**) The left curve is UV-vis absorption of the GQDs-F sample dispersed in water. The right curve is the emission spectrum of GQDs-F under 310 nm excitation. (**b**) The PL spectra of the GQDs-F at different excitation wavelengths. (**c**) The PLE spectrum of the GQDs-F with the detection wavelength of 428 nm (the blue curve) and 533 nm (the green curve).

While fully fluorinated graphene with perfect sturcture is quite stable [28,29], the presence of atomic defects in the fluorinated graphene sheet leads to an obvious lowering of the melting temperature [29]. According to the above XPS analysis, we give the schematic models in Figure 3 to better understand the mechanism for the thermal cutting of FG into GQDs-F. Because of the non-negligible formation of CF_n_ (*n* = 2, 3) in FG, it can be deduced that FG is highly fluorinated graphene with a great number of structure defects (shown in the left of Figure 4). In the annealing of FG, the rupture is not only for C-F bonds, while at the lattice defects of the graphene sheet, the C–C bond is much weaker in CF–CF_n_ (*n* = 2, 3) structure and is easier to be broken, leading to the evaporation of CF_n_ fragments [30,31]. At the same time, many F-atoms break the original local bonds in the annealing. Instead of being detached from the graphene, they transfer to the edges of the structure defects and then detach from the sheet with the bonded C atoms [29]. That is, the presence of these structure defects makes the FG fragile and readily attacked. Like scissors, the CF_n_ (*n* = 2, 3) groups near carbon vacancy defects lead to the loss of around skelton C atoms in annealing, and greatly enlarge the structure defects of FG. When the annealing temperature is high enough (>780 K), FG can be completely cut into scattered graphene quantum dots (shown in the right of Figure 4). Because of the high stability of fluorine atoms at graphene defective sites and edges [35], remanent fluorine adatoms should be mostly like to bond at the edges of GQDs-F. Otherwise, the residual oxygen containing groups may locate at the edge or the basal planes. We have also undertaken the thermal annealing at 680 K, and explore the defect forming at a low annealing temperature (Figure S2, Supplementary Materials). It is found in Figure S2 that FG did not decompose into grahene nanodots when annealed at 680 K, and the sample FG-680 maintain the two-dimensional flexible structure because of only part of CF_n_ groups of FG detached from graphene skeleton. Such novel synthesis of GQDs-F is highly effective and convenient. The weight of GQDs-F is 13.2 wt % weight of the raw materials FG, indicating that it is a high-yield approach. 

Figure 4 gives the optical properties of GQDs-F. As shown in Figure 4a, the Uv-vis absorption spectrum of GQDs-F displays a typical absorption band at around 300 nm, which indicates π–π* transition of aromatic sp^2^ domains [10]. A detailed PL behavior was carried out under different excitation wavelengths. As shown in Figure 4b, when the excitation wavelength is varied from 300 to 420 nm, the PL peak shifts to longer wavelengths, and GQDs-F demonstrate bright blue emissions, with the strongest peak position at 428 nm (excited at 310 nm). When the excitation wavelength changed from 440 to 500 nm, the green emission peaks vary from 515 to 545 nm, and there is another maximum emission peak that is centered at 533 nm (excited by 480 nm), showing obvious excitation wavelength dependence. Such fluorescence of long wavelength of GQDs is quite favorable for bioimaging. In brief, the GQDs-F afford multicolor fluorescent emission depending under different excitation wavelengths. The PL quantum yield of GQDs-F was tested to be 6.30% by use of quinine sulfate as a standard at 310 nm excitation, which is slightly less than that of previously reported GQDs [9]. It could be due to the low fluorination that can somewhat induce the PL quenching because of the higher electronegativity of F. Figure 4c gives the PL excitation (PLE) spectrum corresponding to the strongest luminescence, which shows two sharp peaks. They can be regarded as the two sorts of the electronic transition, which is from σ and π orbitals of sp^2^ triple carbenes to the lowest unoccupied molecular orbital (LUMO) [21,24]. The electronic transitions detected are 262 nm (4.73 eV) and 310 nm (4.0 eV) when the emission wavelength λ_em_ = 428 nm, and 375 nm (3.31 eV) and 480 nm (2.58 eV) when λ_em_ = 533 nm. The energy difference of σ and π orbitals can be calculated to be ca. 0.73 eV, which are probable energy difference for triplet ground state of carbene (<1.5 eV).

The most accepted mechanism of the PL for GQDs is considered as the size effect [10]. The strong blue emission arising from GQDs-F can be defined as an intrinsic emission stemming from the most preferential isolated sp^2^ subdomain, and this emission mode has been proved as the primary blue emission from minimally oxidized GQDs [10,36]. However, the appearance of second PL peak with green emission may be due to the coexistence of a little larger sizes of isolated sp^2^ subdomains with lower energy gaps [9]. Small conjugated subdomains with various sizes may coexist in GQDs, and have different energy gaps, which can result in the split of the PL emission spectrum [9,10]. Besides the intrinsic energy level of π and π*, there are various emissive traps induced by structural defects and lowly doping of fluorine and oxygen on the surface of GQDs-F [10]. The emissive traps generate different discrete energy levels, and thus can promote excitation wavelength dependence, which further leads to colorful PL emissions under different excitation energies. When compared to the fluorinated GQDs prepared by hydrothermal method with high fluorinated and oxidized degree [21,22], the maximum PL peak of the GQDs-F show a clear blue-shift of ca. 10 nm in the PL emission spectra. It is because the prepared GQDs-F with a smaller number of heteroatom has less additional states of sp^3^ carbon acting as emissive traps, leading to a possibility of blue-shift in the PL spectra. 

**Figure 5:**
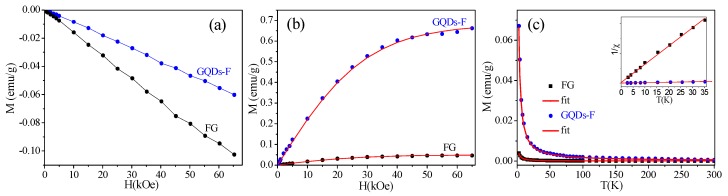
(**a**) The magnetization *M*(*H*) curves of FG and GQDs-F measured at 300 K by SQUID. It is found that both the samples exhibit linear diamagnetism at 300 K; (**b**) The initial *M*(*H*) of FG and GQDs-F measured at 2 K. Symbols are measured data, and the solid curves are fitted curves of Brillouin function; (**c**) The *M*(*T*) curves of FG, GQDs-F under an applied field of 3 kOe. Inset is the corresponding 1/*χ* (*T*), and the solid lines are fitted lines of the Curie law.

Subsequently, we carried out the magnetic measurements for FG and GQDs-F. It is found that only linear diamagnetism is observed in the samples at 300 K (Figure 5a). Figure 5b gives the dependence of magnetization *M* (after deducting the corresponding diamagnetic characteristic) on applied field *H* at 2 K. The *M*(*H*) curves of FG and GQDs-F fit well with the Brillouin function [17,18]

(2) $$M=M_{s}\left[\frac{2J+1}{2J}Coth\left(\frac{2J+1}{2J}x\right)-\frac{1}{2J}Coth\left(\frac{x}{2J}\right)\right]$$

where saturation magnetization $M_{S}=NgJ\mu_{B}$, $x=gJ\mu_{B}H/\left(k_{B}T\right)$, *g* is the *g*–factor (assuming *g* = 2), *J* is the angular momentum number, *N* is the number of spins and *k_B_* is the Boltzmann constant. The *M_s_* of FG is fit to be 0.049 emu/g with *J* = 0.6, and the *M_s_* of GQDs-F is enhanced to 0.680 emu/g with *J* = 0.5. It has been found that the GQDs-F exhibit a typical spin-half paramagnetism induced by point defects [17,18]. It is worth noting that *J* value of FGO is not be integral multiple of 1/2, and can be explained as an average over all the sample with uneven magnetic structure. Figure 5c shows the dependence of *M* of FG and GQDs-F on temperature *T* when *H* = 3 kOe, respectively. Inset are the corresponding 1/*x*–*T* curves, which fit the Curie law curves $\chi =NJ(J+1)g^{2}\mu_{B}{}^{2}/(3k_{B}T)$ well, corroborating purely Curie–like paramagnetic behavior. Note that after the magnetic measurements, ICP measurement was carefully performed to exclude the magnetic contribution of metals. The magnetic impurity of the samples was confirmed to be below 25 ppm, contributing to negligible magnetic signal (Table 2). 

Clearly, the magnetism of the prepared samples originates from their intrinsic structure. As is known, a stoichiometric derivative of graphene with each carbon attached to fluorine atom is intrinsically nonmagnetic due to the absence of unpaired spins [17]. However, the prepared FG sample with a high F/C atom ratio of 1.30 still have a weak paramagnetism (*M_s_* = 0.049 emu/g), attributing to a small portion of sp^2^-conjugated carbon (2.4%) that is embedded in the sp^3^ matrix of FG [18]. More interestingly, after the FG was cut into GQDs, the sample has greatly enhanced magnetism. Extracted from the Brillouin fits in Figure 5b, the number of localized spins of GQDs-F is calculated to be ca. 7.33 × 10^19^ g^−1^. As reported, the magnetic moments of GQDs are from vacancies, sp^3^-type defects, and magnetic zigzag edges [12,13,14]. Based on the above XPS analysis, there is 3.0 percents of remanent F adatoms, which are prone to be located at the edge of GQDs-F. For local magnetic moments arising only from the edges of F clusters on graphene [17,18], the sprinkling of F adatoms located at the edge of GQDs-F have a high efficiency of introducing paramagnetic centres. Additionally, there are few carbonyls groups in GQDs-F, so the carboxylation of GQDs’ edges without magnetic contribution can be suppressed [15]. In fact, the exact edge structure that is obtained by top-down methods includes not only the prior zigzag edges, but also armchair edges and other types of edge [37]. Although the bare zigzag edges were reported to be inclined to reconstruct into non-magnetic edge type [38], residual zigzag edges with the σ bonds passivated by adatoms can be kept from reconstruction and preserve the edge states magnetism [16,39]. Assuming that all of the F adatoms bond at zigzag edges and induce spin-half paramagnetism, the number of localized spins *N* can be calculated from the atom ratio of GQDs-F to be ~128.89 × 10^19^ g^−1^, which is much higher than that obtained from the *M_s_* (~7.33 × 10^19^ g^−1^). It indicates that many F adatoms also bond at non-zigzag edges without magnetic contribution, or some still scatter as small F clusters on basal plane of GQDs-F. It is found that the number of localized spins of GQDs-F (~7.33 × 10^19^ g^−1^) is larger than the values (5.28 × 10^19^ g^−1^) of the GQDs with an average small size (ca. 2.04 nm), as reported in Ref. [16]. That is, GQDs-F have a lower edge-to-area ratio and a smaller percent of edge atoms, but have a higher localized spin density. Hence, the higher magnetic moments of the GQDs-F indicate that there are more residual zigzag edges with the σ bonds passivated by remanent F adatoms, which can preserve the magnetic edge states. Certainly, the point vacancies or the few residual hydroxyl groups, which may be located at the edge or basal planes, can also contribute to magnetism of GQDs-F. Notably, although the *M_s_* of GQDs-F is relatively high, there is no sign of magnetic ordering exiting in the system because of still many magnetic edge states suppressed by the reconstruction [16].

## 4. Conclusions

In conclusion, we develop a novel approach of slightly F-doped GQDs. The raw materials FG have a number of structure defects, characterizing by a high percent of C–F_n_ (*n* = 2, 3) bonds. Different from the unzipping mechanism of graphene into GQDS through hydrothermal route, the CF_n_ (*n* = 2, 3) groups near structure defects are like scissors in our method, which can evaporate as C_2_F_4_ product and convert FG into GQDs-F in pyrolysis. The GQDs-F sample has an average diameter of 5.11 nm (extracted from TEM images), and a low F/C atomic ratio of 0.03. Because of the lowly doping of fluorine and oxygen, the GQDs-F show an intrinsic blue emission and excitation wavelength-dependent properties, exhibiting a clear blue-shift in the PL spectra when compared to those of GQDs that are prepared by other methods with a higher fluorinated and oxidized degree. Additionally, it is found that GQDs show a spin-half paramagnetism (*M_s_* = 0.680 emu/g), and the number of localized spins of GQDs-F is high up to ca. 7.33 × 10^19^ g^−1^. It is because there are a lot of residual zigzag edges with the σ bonds passivated by remanent F adatoms, which are kept from reconstruction and preserve the magnetic edge states. The single materials GQDs-F with multimodal properties, such as multi-color PL property and strong paramagnetism, must expand their practical application field.

## Figures and Tables

**Table 1:** The locations and contents of chemical groups in the samples determined from the integral intensities of C 1s XPS peak.

Chemical Groups	C–C	C–CF, C–OH	C–O	C=O	CF	CF_2_	CF_3_
Location (eV)	284.5	286	286.7	288.2	289.5	291.2	293
FG (%)	2.4	2.1	2.9	4.4	55.9	23.9	8.4
GQDs-F (%)	82.9	6.4	4.2	3.5	3.0	0	0

**Table 2:** The contents of the metal impurity elements of FG and GQDs-F measured by the ICP spectrometry. The unit is ppm, and ‘ND’ denotes ‘not found’.

Samples	Fe	Co	Ni	Mn	Al
FG	8.5	ND	ND	13.0	ND
GQDs-F	9.5	ND	ND	15.0	ND

## Supplementary Materials

The following are available online at www.mdpi.com/1996-1944/11/1/91/s1, Figure S1: The size distribution of QGDs-F extracted by analysis of DLS data, Figure S2: The typical TEM images and C 1s XPS spectra of FG-680.
