# Supplementary material for: Novel Synthesis of Slightly Fluorinated Graphene Quantum Dots with Luminescent and Paramagnetic Properties through Thermal Cutting of Fluorinated Graphene
Source: Materials (Basel). 2018 Jan 8;11(1):91. doi: 10.3390/ma11010091 (PMC5793589; doi:10.3390/ma11010091)
Supplement: Supplementary file 1 [file materials-11-00091-s001.pdf]

Supplementary data

Novel synthesis of slightly fluorinated graphene quantum dots with luminescent and paramagnetic properties through thermal cutting of fluorinated graphene

Qian Feng [1,2,*], Wenqing Xiao [1,2], Yuan Liu [3], Yongping Zheng [1,2], Yuda Lin [1,2], Jiaxin Li [1,2], Qingying Ye [1,2] and Zhigao Huang [1,2,*]

[1] Fujian Provincial Key Laboratory of Quantum Manipulation and New Energy Materials, College of Physics and Energy, Fujian Normal University, Fuzhou 350117, China; wqxiao1995@163.com (W.X.); zyp@fjnu.edu.cn (Y.Z.); linyuda1993@163.com (Y.L.); lijiaxin@fjnu.edu.cn (J.L.); qyye@fjnu.edu.cn (Q.Y.)

[2] Fujian Provincial Collaborative Innovation Center for Optoelectronic Semiconductors and Efficient Devices, Xiamen 361005, China

[3] Faculty of Science, Jiangsu University, Zhenjiang 212013, China; yliu@ujs.edu.cn

* Correspondence: fengqian@fjnu.edu.cn (Q.F.); zghuang@fjnu.edu.cn (Z.H.); Tel./Fax: +86-591-22867577 (Z.H.)

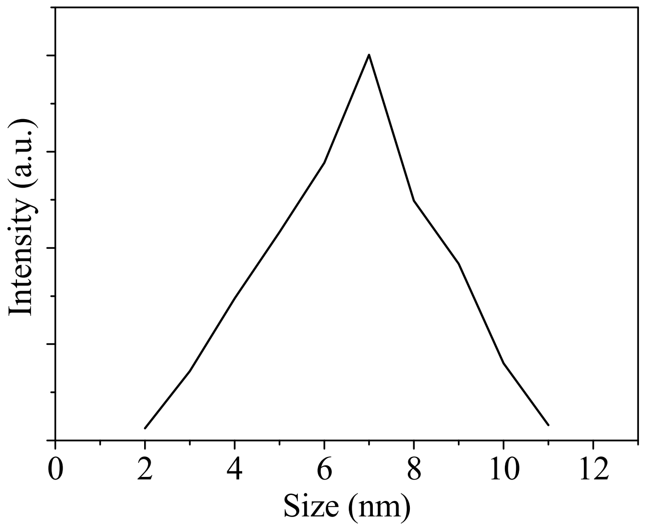

Figure S1. The size distribution of QGDs-F extracted by analysis of DLS data.

The corresponding DLS result provides further demonstration of small and relatively uniform sizes of the resultant QGDs-F (average hydrodynamic diameter ~6.64 nm), as is shown in Fig. S1.

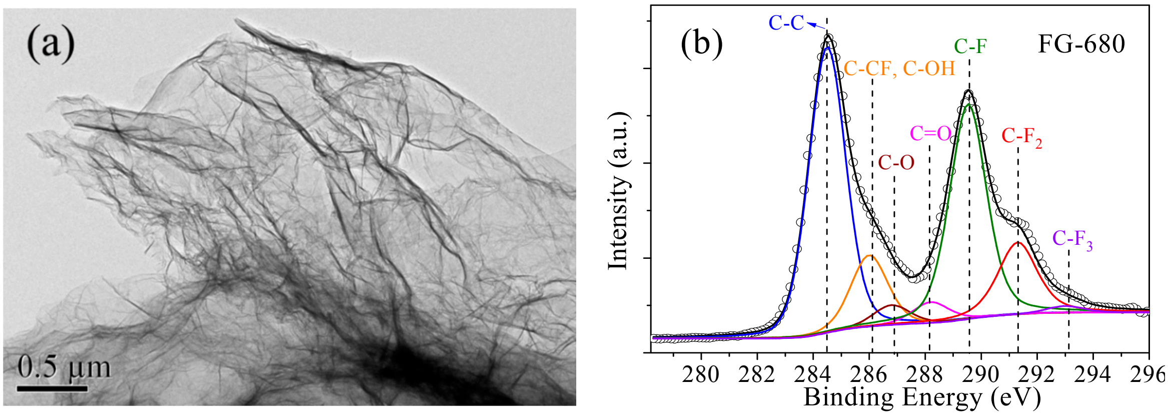

Figure S2. The typical TEM images and C 1s XPS spectra of FG-680.

Figure S2 gives the typical TEM images, and C 1s XPS spectra of FG-680. Different from that of FG-780 and FG-810, it is seen that the sample FG-680 maintain the two-dimensional flexible structure with many wrinkles. It is seen that the C 1s spectrum of FG-680 was deconvoluted into several components, and there are still residual CF_n (n=2, 3) of 13.0%, indicating that not all CF_n groups of FG detached from graphene skeleton. Hench, FG didn't thermally decompose into scattered grahene quantum dots at 680 K. The carbon loss calculated from C atomic percents and the residual mass of FG-680 is 20.7 %, which is larger than the drop of CF_n (n=2, 3) bonds (19.3%). It can be reasonably speculated that the defluorinated sample FG-680 has many structure defects, and the loss of skelton C atoms is because of not only the preferable break of CF-CF_n (n=2, 3) bonds to CFn fragments, but also the producing of some new C vacancies on the basal graphene plane.
